# Approved Societies in Relation to Voluntary Hospitals

**Published:** 1924-07

**Authors:** R. J. Meller


### APPROVED SOCIETIES IN RELATION TO VOLUNTARY HOSPITALS.

By R. J. MELLER, M.P.

I represent a group of societies comprising over one-half of the insured population; and, although I have no authority to speak for societies not within that group, I know from personal experience that the work of your hospitals is highly appreciated by them, and that there is the keenest desire to co-operate with you to the fullest possible extent. Quite early in the career of the Approved Societies it became evident that the medical necessities of an insured population could not be met at the hands of the private practitioner only, and even to-day—twelve years after the coming into operation of the Insurance Act—we find a clamant demand for the extension of medical service.

### Striking Figures.

I find in the Cave Report of 1921 that in the Metropolitan Police district there are 117 hospitals, the rest of England and Wales 728, and in Scotland 107, making a total of 952. The beds available are—in London—approximately 12,797; England and Wales, 31,265; Scotland, 8,132; in all, just over 52,000 beds. There are roughly 15 million insured people for whom you must be prepared to cater, apart altogether from the women and children who do not come within the scheme of National Health Insurance. The only other figure I want to quote from the Cave Report is that the estimated deficiency in the revenue required for the proper upkeep of the hospitals in Great Britain for 1921 was £1,000,000. These were striking figures and could not be ignored by the Approved Societies. If the contributions from Approved Societies can increase the number of beds available or reduce the deficiency, there is ample justification for the support which is now being given.

### The Societies' Problem.

When the surpluses of the Societies became known you were passing through a very difficult time, due largely to the shortage of money, and also—a fact, of course, most important from your point of view—the increased cost of maintaining your institutions. The Approved Societies were not unaware of the difficulties through which you were passing, for they had from day to day evidence that many of their members in a serious condition, were unable to obtain hospital treatment—not in every case because there was lack of accommodation, but because, owing to the depletion of your funds, many of the beds had to be closed. The Societies had no easy problem before them, because their members were finding that the cost of living had risen and was rising. Similarly, the expenses of illness were greater than in pre-war days, and they naturally looked to the Societies, if it was in their power, to help them by an increased sickness allowance. Some Societies were so placed that they were able to offer, not only additional cash benefits, but also other benefits in kind, including a contribution towards the cost of hospital treatment.

### The Cave Committee Report.

It was perhaps a fortunate thing that just about the time when you were seeking for further sources of support, the report of the Cave Committee, to which I have made reference, was issued, and the information obtainable from that report is of the greatest importance in the consideration of your position to-day. We were told "If any considerable number of beds closed down, the shortage of accommodation would be such that the public would be compelled to step in and supply the deficiency." It was suggested by a small minority of the witnesses that liability for the hospitals should be taken over by the State and thrown upon the rates. "In our view," said the Commissioners, "either proposal would be fatal to the voluntary system. . . ." It may be said by some critics that the position the Approved Societies should have taken up in these circumstances, seeing that they were part of a national scheme, was to refrain from contributing their funds to support a voluntary institution; but if the Societies had any doubts in this connection, I think their minds must have been influenced by the reply of the Cave Commission to the question, "Is the voluntary system worth saving?" Their answer was very decided: "We are convinced that it is." And if I may respectfully say so, I think the reasons for their decision are unanswerable.

### Support of Hospitals.

Payment by the Societies is summed up in the following words: "While we hold that the Approved Societies are not under any obligation legally or equitably to provide the whole cost of the maintenance and treatment of their members in hospital, we are strongly of opinion that they do owe to the hospitals a large measure of support." With this statement I do not propose to disagree, and I think that my remarks are an acceptance of the view of the Commission. It was suggested in the report that "The clerical labour involved in distributing any contribution from insurance funds among the hospitals concerned will be considerable."

### A Satisfactory Scheme.

I agree that at one time it was thought this would be the case, but the scheme which has been devised is, I believe, a simple, economical and satisfactory one. It does provide that according to the assistance rendered by the hospital to the insured patient, so shall the hospital receive its due proportion of the fund available. An ideal system would be a full and adequate payment in respect of each week's treatment given, but I fear this happy position is not likely to arrive in the very near future. The group of Societies with which I am connected was able during the year 1923 to distribute the sum of £170,000 to the voluntary hospitals, and I confidently anticipate that a similar sum will be available for the year 1924.

